# 循环肿瘤细胞在肺癌中的研究进展

**DOI:** 10.3779/j.issn.1009-3419.2012.11.03

**Published:** 2012-11-20

**Authors:** 慧 李

**Affiliations:** 130012 长春，吉林省肿瘤医院胸部肿瘤内一科 Department of Thoracic Medical Oncology, Jilin Provincial Cancer Hospital, Changchun 130012, China

**Keywords:** 肺肿瘤, 循环肿瘤细胞, 检测, Lung neoplasms, Circulating tumor cells, Testing

## Abstract

肺癌是我国发病率及死亡率最高的恶性肿瘤，其早期诊断和监测复发与转移，对提高肺癌5年生存率尤为重要。循环肿瘤细胞（circulating tumor cells, CTCs）作为一种代表原发肿瘤的“液态活检标本”，有助于实时、无创性地进行组织学鉴定。CTCs检测有助于肺癌早期诊断、监测肿瘤复发与转移、判定疗效和预后，而且可能成为检测临床分子、分析耐药分子机制及解决肿瘤异质性的一种手段。本文就近年来CTCs在肺癌领域的研究进展进行综述。

## Abstract (en)

Lung cancer is the malignant disease with the highest rate in terms of incidence and mortality in China. Early diagnosis and timely monitoring tumor recurrence and metastasis are extremely important for improving 5-year survival rate of lung cancer patients. Circulating tumor cells (CTCs), as a " liquid biopsy specimens" for the primary tumor, provide the possibility to perform real-time, non-invasive histological identification for lung cancer patients. The detection of CTCs contributes to early diagnosis, surveillance of tumor recurrence and metastasis, and prediction of therapeutic efficacy and prognosis. Furthermore, CTCs-dependent detection emerges as a new approach for molecularly pathologic examination, study of molecular mechanisms involved in drug resistance, and resolution for tumor heterogeneity. This study reviewed the recent progress of CTCs in lung cancer research field.

肺癌是我国发病率及死亡率最高的恶性肿瘤，手术、化疗、放疗及分子靶向治疗使肺癌的治疗有了很大的提高，但其5年总生存率仍徘徊在10%-15%。源于肺癌肿瘤细胞的不断增殖，通过上皮-间质转化（epithelial-mesenchymal transition, EMT）等形态学变化，细胞会分泌胞外酶降解细胞外基质（extracellular matrix, ECM），造成细胞间及细胞与周围基质间相互附着力下降，从而利于肿瘤细胞逃离组织束缚进入循环系统，成为具有侵袭转移能力的CTCs^[1]^。近年来随着肿瘤的研究和治疗技术的提高，发现许多肿瘤患者虽然在治疗后临床上检测不到残余的肿瘤细胞，但最终患者因肿瘤复发和转移而死亡，这可能与单独用影像学技术不能检测到微小残留肿瘤细胞有关。这些细胞存在于外周血、骨髓、淋巴结中，在一定条件下增殖浸润，引起肿瘤复发、转移。这些复发、转移的肿瘤细胞表型和表达谱与原发灶相似，因此CTCs作为一种代表原发肿瘤的“液态活检标本”为肺癌患者实施实时、无创性检测提供了资源。而且研究^[2-6]^发现检测CTCs有助于肺癌的早期诊断、监测复发与转移、判定疗效、制订个体化治疗方案及判断预后。

目前随着对肺癌驱动基因、癌基因、抑癌基因等基因组学的研究以及对肿瘤生物标志物表达、缺失、差异性表达的研究，使肺癌治疗进入了针对分子标志物化疗、靶向治疗的时代。但目前我们面临的是如何提高药物疗效并减少毒性反应、如何克服耐药性以及解决肿瘤异质性问题，这些难题的攻克均需要我们提供组织标本。但单次组织活检由于受肿瘤的基因组变异引起的异质性影响，可能会造成个体化诊断和分子标志物分析的偏差。特别是在肿瘤的进展转移过程中，如何检测与转移或耐药相关的新的分子变异很重要。目前开展实时活检在临床中难以被患者接受，但随着基因组技术的进步，特别是CTCs的研究进展，未来理想有可能变为现实。

## 1 CTCs的检测方法

尽管在多种肿瘤患者的外周血中能够检测到CTCs，但总体上来说，血液中CTCs的数量非常少，而且在不同肿瘤患者之间或是同一肿瘤的不同患者之间CTCs数目具有较大差异。目前的主流理念是先对外周血中的肿瘤细胞进行富集，然后对其进行检测。富集方法主要是利用肿瘤细胞独特的表面标志或物理特性来进行。后续的检测包括基因水平的检测或细胞水平的检测，其中基因水平检测的方法主要是实时定量反转录PCR（quantitative reverse-transcription PCR, qRT-PCR），细胞水平的检测方法有流式细胞检测技术（fluocytometry, FCM）、Cellsearch、CTC芯片（CTC-chip）和膜滤过分离肿瘤细胞技术（isolation by size of epithelial tumor cells, ISET）等。各种方法各有其优缺点。

### 1.1 qRT-PCR法

秉承了PCR技术的灵敏度，可在1×10^7^个-1×10^8^个外周血细胞中检测出单个肿瘤细胞，但该方法是通过肿瘤标志物的含量推测CTCs的数目，并不能真实反映CTCs的个数。因为不同的CTCs之间肿瘤标志物的表达水平不同，因而肿瘤标志物的含量与肿瘤细胞的实际个数并不总是具有正相关性^[7]^。

### 1.2 FCM法

采用荧光标记相应抗体来检测CTCs，其优点是操作简单、快速、数据精确。缺点是FCM不能提供细胞形态方面的信息，无法区分带有同样标志物的肿瘤细胞和正常细胞。由于FCM检测靶细胞的敏感度仅为1/10万，而外周血中肿瘤细胞的数量常少于1/100万，因此该方法的价值在很大程度上依赖于可分析的细胞数量，极大地限制了该技术的广泛应用^[8]^。

### 1.3 Cellsearch法

Cellsearch系统是将细胞富集后再进行自动化检测，其生物学原理是依据肿瘤细胞与正常细胞表达的表面蛋白不同而将二者区分开来。来源于中胚层的白细胞表达CD45，来源于外胚层的癌细胞表达上皮细胞粘附分子（epithelial cell adhesion molecule, EpCAM）和细胞角蛋白（cytokeratin, CK），但不表达CD45。该系统首先通过正选择将血液中表达EpCAM的细胞分离出来，然后进行免疫荧光染色，将EpCAM^+^CK^+^DAPI^+^CD45^-^的细胞界定为CTCs。应用Cellsearch系统可以检测7.5 mL的血液样本（约含400多亿的血细胞）中单一的CTCs，因此具有敏感度高、可操作性强、高度自动化检测、数据精准等优点。但该检测系统会遗漏缺失上皮细胞表面标志的肿瘤细胞，例如经过EMT后的间质细胞。此外，在检测团块状的CTCs方面也不具有优势^[9]^。

### 1.4 CTC-chip法

CTC-chip技术的生物学原理与Cellsearch基本相同，也是将CTCs认定为带有EpCAM和CK的表达但不带有CD45表达的细胞群。CTC-chip技术具有更高的灵敏性，可以在1 mL血液中检测出循环肿瘤细胞。第一代的CTCs芯片是基于微流体学原理，在硅片上面覆盖众多的显微位点，每一个位点上都包被着能够捕获CTCs的抗体。当血液样品通过微流芯片时，CTCs就能被捕获到^[4]^。因为第一代的CTCs芯片仅适用于小规模的实验室研究，而不能应用于大规模的临床研究，第二代的CTCs芯片又名herringbone（HB）芯片应运而生。改进之处在于将微芯片安装在标准载玻片上，不但可以利用常规病理检测方法对癌细胞进行鉴别，也加大了样本的处理量并提高了捕获团块状CTCs的能力^[10]^。

### 1.5 ISET法

ISET联合激光扫描细胞计量仪技术的原理是首先利用上皮源性的肿瘤细胞比血液中的其它细胞大的特性，采用孔径为8 μm的滤膜，将肿瘤细胞从血液中分离出来，然后通过荧光标记细胞来进行进一步鉴定，常用的荧光抗体包括抗CK抗体或者抗lineage-specific抗体。采用此方法已成功地在乳腺癌、前列腺癌和非小细胞肺癌（non-small cell lung cancer, NSCLC）患者的外周血中检测出CTCs^[11]^。但采用此方法检测的CTCs数目与采用Cellsearch技术检测的数目之间存在不一致性，可能有假阳性结果出现^[12]^。该方法的优势之一是可以将失去上皮细胞特征的肿瘤细胞分离出来，例如经过EMT的间质细胞等。

## 2 CTCs在肺癌中的临床应用

### 2.1 CTCs与肺癌早期诊断

肺癌的早期诊断对治疗效果至关重要，早期发现并及时治疗是提高生存率的关键。研究^[3, 13]^显示CTCs对肺癌早期诊断具有潜在的应用价值。目前，在无明显临床症状和转移灶的Ⅰ期患者体内也可检测到CTCs，说明现有临床分期的早期就已存在肺癌细胞的播散^[14]^，并能在大量聚集后形成具有高度转移潜能的循环肿瘤微栓。

Allard等^[15]^最先采用Cellsearch系统检测了99例肺癌患者（主要是NSCLC）的168个样品，发现肺癌患者外周血中CTCs数目≥2、≥5、≥10和≥50的患者分别占总患者的20%、14%、10%和6%。随后Nagrath等^[4]^采用CTC-chip检测了55例肺癌患者外周血中CTCs，结果显示所有患者的血液中均检测出5个以上的肿瘤细胞，其中每毫升血液中CTCs数目为5-20、21-50、51-100和 > 100的阳性率分别为20%、20%、20%及40%。2009年后关于肺癌患者外周血中CTCs的论文不断涌现。Wu等^[16]^检测了47例肺癌患者，其中41例为初治患者，6例为复发患者；3例为Ⅰ期-Ⅱ期，22例为Ⅲ期，22例为Ⅳ期；按病理类型可分为腺癌27例，鳞癌7例，小细胞肺癌（small-cell lung cancer, SCLC）13例。结果显示每7.5 mL血液中CTCs数目≥2的患者在初治的Ⅲ期腺癌中为78%，Ⅲ期鳞癌中为75%，Ⅲ期SCLC中为60%，在Ⅳ期腺癌和Ⅳ期SCLC中，CTCs阳性率分别为46%和71%，复发的患者CTCs阳性率高达83%。

### 2.2 CTCs与肺癌复发、转移

肺癌的复发、转移是造成患者死亡的重要原因，而外周血中存活的肿瘤细胞可能是造成肺癌转移和复发的机制之一。因此研究CTCs的生物学特性可以为抑制肺癌的转移和复发提供有价值的理论依据。

日本的Tanaka等^[5]^检测了125例肺癌患者外周血中的CTCs，其中包括85例腺癌，22例鳞癌，9例SCLC，9例其它病理类型的肺癌。结果显示在30.6%的肺癌患者中检测到CTCs，但在12%的非恶性肿瘤患者（*n*=25）中也发现相同表型的细胞，统计学分析结果表明CTCs数目间的差异不足以区分肺癌和非恶性肿瘤。在纳入该研究的患者中，31例伴有远处转移，94例无远处转移。进一步分析发现CTCs水平与肿瘤的远处转移具有相关性，发生转移的患者体内CTCs数目升高，检测出CTCs > 1的敏感性和特异性分别为71%和83%。通过该研究作者认为CTCs是一个有用的、可监测肺癌转移的生物标志物。

### 2.3 CTCs与肺癌疗效

当前对肺癌的治疗是基于肿瘤病理分级、患者一般状况及肿瘤发展趋势等因素之上的综合治疗。但遵循统一的诊断标准建立的治疗方案缺乏个体化。随着对CTCs研究的深入，发现其可反映肿瘤组织基因的表达变化，有助于监测疗效及建立个体化的治疗方案。

Nagrath等^[4]^发现在评估化疗的疗效上，CTCs数量的改变与按照实体瘤的疗效评价标准（Response Evaluation Criteria in Solid Tumors, RECIST）进行计算机断层扫描（computed tomography, CT）检查的结果具有高度一致性。Wu等^[16]^应用Cellsearch对12例化疗2个周期的肺癌患者跟踪随访，也发现CTCs数量与化疗后影像学的应答存在相关性，提示CTCs可实时监测肺癌患者化疗的疗效。

采用CTCs作为生物标志物预测药物疗效的一项Ⅱ期临床试验^[17]^正在进行。该研究纳入了39例复发和耐药的SCLC患者，采用替莫唑胺治疗前，患者CTCs为0-929，7例为0，13例为1-4，19例≥5；存在肝转移的患者较非转移患者CTCs数目更高；出现新转移灶的患者较原有病灶进展的患者CTCs水平高（*P*=0.008）；治疗第1周期末的CTCs数目与采用RECIST进行的疗效评价结果具有相关性（*P*=0.041），CTCs增高的患者总生存期（overall survival, OS）缩短（5个月*vs* 8个月），但是基线CTCs不能预测治疗反应，也不能预测无进展生存期（progression free survival, PFS）。

肺癌组织基因的表达或突变可在治疗过程中发生变化，从而导致药物敏感性的改变，如耐药基因T790M的出现可导致初始对吉非替尼敏感的NSCLC患者的治疗失败。Maheswaran等^[6]^检测了接受吉非替尼治疗的NSCLC患者的CTCs中表皮生长因子受体（epidermal growth factor receptor, EGFR）突变情况，发现其与肿瘤组织具有高度一致性，例如同样存在T790M突变。在治疗过程中监测CTCs，发现CTCs数量的改变可以反映患者病情的变化趋势和治疗的疗效。

### 2.4 CTCs与肺癌预后

在肺癌患者中，CTCs数目与肿瘤进展呈正相关，尤其伴随肿瘤远处转移而升高。因此，对肺癌CTCs研究显示CTCs具有重要的预后价值^[5, 18, 19]^。

英国的Hou等^[18]^检测了50例SCLC患者（20例局限期和30例广泛期）外周血中的CTCs，这些患者都是初次应用化疗药物（如铂类联合依托泊苷），放疗和预防性脑照射在第2个化疗周期开始并与化疗同时进行或是在第6个化疗周期后进行，在第1个疗程的第1天及第15天进行早期疗效评估。化疗前86%的患者中检测到CTCs，经过化疗后患者CTCs阳性率下降至60%。在化疗后第22天时检测到的中位CTCs为1，持续升高的CTCs预示着患者的预后差（*P*=0.01）。进一步对15例样本（CTCs数目为0-5, 884）进行分析后发现，所有被检测出CTCs的样本中均可见CD56阳性细胞。CD56属于神经粘附分子且在小细胞肺癌中高表达，因此该数据说明循环血液中的CTCs具有与组织中原发肿瘤相似的特性，即具有上皮-神经内分泌的双重特征。此外，CTCs的水平与病理分期及功能状态评分相关。广泛期患者的中位CTCs为237，局限期为2，7例未检测到CTCs的患者均为局限期患者。CTCs的数量也与肝转移成正相关，肝转移患者与非转移患者中位CTCs分别为1, 197和4。CTCs水平与患者的生存相关，是一个预后因子，CTCs数目 > 300的患者中位生存时间为134 d，CTCs数目 < 2的患者中位生存时间为443 d（*P* < 0.005）。该研究报道的CTCs阳性率明显高于Allard等报道的阳性率（86% *vs* 20%），作者分析这与SCLC增殖快、转移发生得早及恶性度高有关。随后，该研究小组扩大了检测的人群^[20]^，通过分析97例SCLC患者，发现85%患者外周血中存在CTCs。化疗前CTCs数目≥50的患者和 < 50的患者相比，OS分别为5.4个月和11.5个月（*P*=0.002），这证明基线CTCs数量是SCLC独立的预后因素。通过探索性分析53例患者在治疗前后CTCs数目的变化，研究人员还发现CTCs数目变化是预测生存最明显的变量（在调整了基线CTCs水平和其它的临床预后因子后），化疗1周期后CTCs数目不能降低到50以下的患者预后不良。

对于晚期NSCLC患者，研究人员^[21]^发现Ⅳ期患者血液中CTCs数目为0-146，Ⅲb期为0-3，而Ⅲa期检测不到CTCs。化疗前CTCs数目 < 5和≥5的患者的PFS分别是6.8个月和2.4个月，OS分别是8.1个月和4.3个月，多因素分析结果显示CTCs数目是最强的预测NSCLC总生存期的指标（*P* < 0.001），如果联合参考治疗前（基线）和第1次化疗结束后外周血中CTCs数目，危险比值更高（*P* < 0.001）。Das等^[22]^研究了17例转移性NSCLC患者CTCs中切除修复交叉互补基因1（excision repair cross complementation1, ERCC1）的表达与预后的相关性。入组患者的特征包括：接受过铂类为基础的化疗；CTCs数目≥2；可检测到ERCC1表达。结果显示以ERCC1表达水平1为临界值，无ERCC1表达的患者PFS延长，ERCC1表达水平高的患者PFS缩短（266 d *vs* 172 d, P < 0.02）。

## 3 问题与展望

已有大量临床试验表明，肿瘤细胞脱落、侵袭并进入血液循环是实现肿瘤转移的最初阶段，CTCs具有重要临床意义。但目前CTCs检测的临床应用仍存在诸多问题：①如何提高检测方法和分子标志物的敏感性和特异性；②多少个CTCs能够代表肿瘤病灶的特征；③阴性结果中尚不能完全排除发生播散的阴性表达细胞，部分检测阴性的患者也在近期出现肿瘤复发转移；④检测的合适时间该如何选择，或者对CTCs阳性患者如何综合治疗。此外原发肿瘤细胞进入外周血循环后是否基因表型与原发肿瘤完全一致仍需进一步研究来论证。

虽然CTCs检测存在一系列问题，且对CTCs的研究多集中在CTCs数量分析上，但CTCs作为新的检测手段，其在肿瘤早期诊断、检测复发、转移、判定疗效和预后等方面的重要作用已得到了大量研究证实^[23-25]^。而且有学者开始关注CTCs本身的差别，如CTCs表面的蛋白表达或其基因突变与临床的关系。目前个体肿瘤间生物学特性的差异是个体化治疗的基础，如分子亚型指导下肺腺癌中检测EGFR、磷脂酰肌醇激酶-3（phosphoinositide-3-kinase, catalytic, alpha polypeptide, PIK3CA）、间变性淋巴瘤激酶（anaplastic lymphoma kinase, *ALK*）、*c-Met*等基因变异的个体化治疗。而个体内的瘤内异质性可能是敏感性差异和获得性耐药的原因之一，因此肿瘤异质性是我们目前面临的难题；另外探讨最关键的分子通路和探寻新的分子标志物是未来的研究重点。CTCs被视为替代原发肿瘤“液体活检标本”，可能有助于临床分子检测、分析耐药分子机制及解决肿瘤异质性的问题。
